# Different changes of bacterial diversity and soil metabolites in tea plants-legume intercropping systems

**DOI:** 10.3389/fpls.2023.1110623

**Published:** 2023-03-16

**Authors:** Shuangshuang Wang, Xiaojia Zhang, Xiaojiang Li, Jiazhi Shen, Litao Sun, Shah Zaman, Yu Wang, Zhaotang Ding

**Affiliations:** ^1^ Tea Research Institute, Shandong Academy of Agricultural Sciences, Jinan, China; ^2^ Tea Research Institute, Qingdao Agricultural University, Qingdao, China

**Keywords:** tea plant, mixed intercropping legume, soil nutrients, soil metagenomics, soil metabolome

## Abstract

As an essential agroforestry, intercropping legumes can improve the physical, chemical, and biological fertility of the soil in tea plantations. However, the effects of intercropping different legume species on soil properties, bacterial communities, and metabolites remain elusive. In this study, the 0-20 cm and 20-40 cm soils of three planting patterns (T1: tea plants/mung bean intercropping, T2: tea plants/adzuki bean intercropping, T3: tea plants/mung bean and adzuki bean intercropping) were sampled to explore the diversity of the bacterial community and soil metabolites. The findings showed that, as compared to monocropping, intercropping systems had greater concentrations of organic matter (OM) and dissolved organic carbon (DOC). Notably, pH values were significantly lower, and soil nutrients increased in intercropping systems compared with monoculture in 20-40 cm soils, especially in T3. In addition, intercropping resulted in an increased relative abundance of *Proteobacteria* but a decreased relative abundance of *Actinobacteria*. 4-methyl-Tetradecane, acetamide, and diethyl carbamic acid were key metabolites mediating the root–microbe interactions, especially in tea plants/adzuki intercropping and tea plants/mung bean, adzuki bean mixed intercropping soils. Co-occurrence network analysis showed that arabinofuranose, abundant in tea plants and adzuki bean intercropping soils, showed the most remarkable correlation with the soil bacterial taxa. Our findings demonstrate that intercropping with adzuki beans is better at enhancing the diversity of soil bacteria and soil metabolites and is more weed-suppressing than other tea plants/legume intercropping systems.

## 1 Introduction

Intercropping is advanced as one of the best conventional production practices used in agroforestry ecosystems (Liu et al., 2017). A set of investigations have demonstrated that compared with monoculture, intercropping enhances biological diversity, improves light utilization, boosts crop quality and yield (Zhang et al., 2015; Huang et al., 2022). Tea plant [*Camellia sinensis* (L.) O. Kuntze] is cultivated worldwide as an economic woody crop, and intercropping with other plants such as legume and walnut has been developed and applied in many tea plantations (Wang et al., 2021; Bai et al., 2022; Huang et al., 2022). Previous studies have shown that tea plant–soybean intercropping affects the expression of metabolites associated with amino acid metabolism, particularly glutamate, lysine, and arginine (Duan et al., 2021). That also improves soil nutrients such as organic matter and total nitrogen (Huang et al., 2022). These studies were mainly focused on the effect of intercropping with sole plant species on soil nitrogen and microbial community structure in tea plantations. However, there is a lack of information and knowledge about the effect of mixed cropping of different legume species and tea plants on changes in soil metabolites and their interactions with bacterial communities based on metagenome level.

Soil microorganisms play central roles in maintaining soil function and influence soil productivity and crop yield (Wang et al., 2017). Microorganisms influence soil nutrient turnover by stabilizing and decomposing organic matter, which affects soil enzyme activities (Peng et al., 2015). Previous studies have shown that plant species and intercropping patterns can affect soil properties and microbial communities. For example, tea plant and soybean intercropping increased soil electric conductivity and available P, K, and increased the relative abundances of organic matter decomposers bacteria (Acidobacteriaceae, Rhodanobacteraceae, and Sphingomonadaceae, etc.). More specifically, complex and dynamic interactions between plants and microorganisms have become an imperative aspect of study in agriculture (Sun et al., 2022). In addition, agricultural management practices that shape microbial communities in the field have improved our understanding of how management factors affect crop quality and yield.

In this study, the effects of intercropping tea plants with different legume patterns (mung bean, adzuki bean, mung bean and adzuki bean) were examined in field experiments. We hypothesized that the different tea plants/legume intercropping systems have different effects on changing soil bacterial community composition and metabolite diversity. Therefore, the primary purpose of the study is to 1) explore the effects of tea plant intercropping with different legumes on soil properties; 2) compare the response of bacterial community composition and metabolites to intercropping with different species of legume; 3) determine the relationship between soil bacterial communities and soil metabolites, and select the higher performance intercropping pattern.

## 2 Materials and methods

### 2.1 Sample collection

The sampling site was in Rizhao, Shandong Province, China (N 35°40′, E 119° 51′). The brown and loamy soil was sampled in Nov. 2021 when the beans were harvested. Field experiments were conducted with four treatments which consisted of tea plant monocropping (CK), tea plant/mung bean intercropping (T1), tea plant/adzuki bean intercropping (T2), tea plant/mung bean, and adzuki bean mixed intercropping (T3). Five soil core samples were collected from each plot using a soil sampler and mixed as one soil sample per plot. Each sample was made up of two depths: 0-20 cm (CK-20, T1-20, T2-20, and T3-20) and 20-40 cm (CK-40, T1-40, T2-40, and T3-40). Each treatment has three replications.

A total of 24 samples were collected. The soils were homogenized and sieved through a 2 mm wire mesh. Then one part of the soil was used for measurement of soil properties, and other parts for metabolome and metagenome sequencing at Wuhan Metware Biotechnology Co., Ltd. (Wuhan, China).

### 2.2 Determination of soil properties

Soil pH was determined using pH strips with 1:5 (wt/vol) soil to water. Soil ammonia nitrogen (NH_4_-N) and nitrate nitrogen (NO_3_-N) were extracted with 2 M KCL. Available phosphorus (AP) was assessed using the ascorbic acid reductant method, and available potassium (AK) was determined through atomic absorption. Total nitrogen (N) concentration was analyzed using a high-temperature reactor to fully combust each sample (Ma et al., 2017). Total phosphorus (TP) and total potassium (TK) were determined by the NaOH molybdenum antimony colorimetric method. The organic matter (OM) was determined using a total organic carbon analyzer (multi-N/C 3100, Jean, Germany). Dissolved organic carbon (DOC) was extracted with 0.5 M K_2_SO_4_ and measured on a TOC analyzer.

### 2.3 Soil metabolome analysis

After homogenization, approximately 0.5 g soil samples with a solution of 1 mL methanol/isopropanol/water (3:3:2, v/v/v) were vortexed for 3 min and ultrasound for 20 min. Supernatants were collected by centrifugation at 12 000 rpm under 4°C for 3 min and then transferred into a sample vial which contained 20 μL ribitol (10 μg/mL, Sigma, St.Louis, MO, USA) as an internal standard to evaporate under nitrogen flow. The resulting extract was incubated in 0.1 mL of methoxy amination hydrochloride in pyridine (0.015 g/mL) at 37°C for 2 h and then in 0.1 mL BSTFA (with 1% TMCS) for 30 min at 37°C. The derivatization solution was added n-hexane to dilute to 1 mL and filtered with a 0.22 μm organic phase syringe filter.

The resulting solution (1 μL) was analyzed by an Agilent 8890 gas chromatograph system coupled with a 5977B mass spectrometer (Agilent Technologies, Palo Alto, CA, USA). Metabolite separation was performed using a DB-5MS column (30 m length × 0.25 mm inner diameter, 0.25 μm film thickness, J&W Scientific, USA). A 1μL aliquot of the analyte was injected in the front inlet mode with a split ratio of 5:1. Helium was used as a carrier gas at a flow rate of 1.2 mL/min. The initial temperature was held at 40°C for 1 min and then raised to 100°C at 20°C/min, raised to 300°C at 15°C/min, and held at 300°C for 5 min. The mass spectrometry data were acquired in full-scan mode. The ion source and transfer line temperatures were 230°C and 280°C, respectively. The relative abundances were calculated based on the peak area and compared to the internal standard.

### 2.4 DNA extraction, library construction, and metagenomic sequencing

Soil DNA was extracted from 0.5 g soil using the EZNA^®^ Soil DNA Kit (Omega Biotek, Inc., Norcross, GA, USA) according to the manufacturer’s instructions. The concentration of DNA was measured using the NanoDrop 2000-UV spectrophotometer (Thermo Scientific, Waltham, MA, USA), and the quality of DNA was monitored on 1% agarose gels.

For the library construction, a total amount of 1 μg DNA per sample was used. Sequencing libraries were generated using NEBNext^®^ Ultra™ DNA Library Prep Kit for Illumina (NEB, USA) following the manufacturer’s recommendations. Briefly, the DNA samples were fragmented by sonication to a size of 350 bp. DNA fragments underwent end-polished, A-tailed, and ligated with the full-length adaptor for Illumina sequencing with further PCR amplification. Finally, PCR products were purified (AMPure XP system, Beckman Coulter, Brea, CA, USA), and libraries were analyzed for size distribution by Agilent2100 Bioanalyzer (Agilent Technologies, Palo Alto, CA, USA) and quantified using real-time PCR. After cluster generation of the index-coded samples, the library preparations were sequenced on an Illumina NovaSeq platform, and paired-end reads were generated.

### 2.5 Metagenome assembly, gene prediction, and functional annotation

To obtain high quality clean data for subsequent analysis, the raw data from the Illumina PE150 platform were trimmed using Readfq (V8, https://github.com/cjfields/readfq). Briefly, sequences with low quality bases (score < 38 in 40 bp length), N base reached 10 bp length, and overlap with adapter sequences above 15 bp were removed. The obtained clean data were assembled and analyzed by MEGAHIT software (v1.0.4), then interrupted the assembled Scaftigs from the N connection and left the Scaftigs without N. The Scaftigs with a length longer than 500 bp were used to predict the open reading frame (ORF). The core-pan gene analysis, basic information statistics, and correlation analysis are based on the abundance of genes among different samples (Shi et al., 2021).

### 2.6 Statistical analysis

The soil properties data were assessed by one-way analysis of variance (ANOVA) with Duncan’s test using SPSS 26.0 (IBM SPSS Inc., United States). Differences were considered statistically significant at p < 0.05. The DIAMOND software (v0.9.9) was used to blast the unigenes to the NR database (including bacteria, fungi, archaea, and viruses) and functional database. The annotated gene numbers, general relative abundance situation, and heat map were counted based on each taxonomy hierarchy’s abundance table. The LEfSe analysis of the functional difference between groups was performed based on functional abundance. The correlation of metabolite and metabolite was calculated by R language, and *p*-values adjusted the statistical test. Spearman’s rank correlation was computed between VIP scores of metabolites and significantly different bacterial populations.

## 3 Results

### 3.1 Changes in soil properties in different tea plant/legume intercropping systems

Some physical and chemical properties were measured to get an overview of the effect of different intercropping patterns on soil (**Table 1**). In 0-20 cm soils, a significantly higher concentration of AK and TK was detected in T3, followed by T2 and T1. T2 and T3 recorded considerably higher DOC compared to other intercropping systems. Though intercropping systems enhanced the concentration of NO_3_-N, AP, and TN, the differences showed no significance.

**Table 1:** Physical and chemical properties of soil.

Soil properties	0-20cm				20-40cm				
	CK-20	T1-20	T2-20	T3-20	CK-40	T1-40	T2-40		T3-40
pH	6.18±0.28	6.03±0.29	6.11±0.45	5.78±0.32	6.5±0.12a	6.44±0.15a	5.91±0.32b		5.95±0.17b
NO_3_-N (mg/kg)	3.84±0.28	4.28±0.96	4.14±0.60	4.56±0.85	4.12±0.61	3.82±0.22	5.09±0.38		5.01±1.09
NH_4_-N (mg/kg)	1.92±0.35	1.99±0.35	1.40±0.08	1.45±0.33	1.65±0.21	1.97±0.89	1.92±0.41		1.81±0.33
AP (mg/kg)	18.94±1.43	21.21±3.93	19.23±1.38	21.71±2.90	15.80±0.80	18.11±2.25	16.78±0.98		17.07±0.80
AK (mg/kg)	92±4.08b	97.67±2.87ab	105.00±15.94ab	118.00±8.52a	118.67±2.87ab	124.00±2.83a	98.33±10.34b		120.67±8.99ab
TN (g/kg)	0.54±0.06	0.52±0.02	0.54±0.05	0.60±0.07	0.35±0.06	0.35±0.07	0.45±0.07		0.44±0.02
TK (mg/kg)	21.13±1.33ab	21.43±1.07ab	20.41±1.08b	23.49±0.66a	21.81±0.71	22.13±0.70	20.83±1.97		21.85±1.59
TP (mg/kg)	0.54±0.07	0.48±0.01	0.47±0.07	0.50±0.03	0.47±0.11	0.43±0.07	0.50±0.11		0.47±0.06
OM (g/kg)	8.36±0.37	8.54±0.47	8.07±0.65	8.01±0.50	5.25±0.65b	7.09±0.69ab	7.48±0.79a		6.13±0.35ab
DOC (mg/kg)	27.76±3.95ab	18.69±0.95b	48.04±6.93a	45.36±3.08a	17.68±0.24c	91.79±9.32a	48.01±4.84b		75.62±8.83ab

AK, available potassium.

AP, available phosphorus.

T, total.

OM, organic matter.

DOC, dissolved organic carbon.

Different letters following data within the same column indicate significant differences at P < 0 .05.

Different from 0-20 cm soil, the content of AK and DOC in 20-40 cm soil in T1 and T3 is higher than in T2 and monoculture. The pH values were significantly lower in T2 and T3 than in T1 and CK. The results indicate that T3 may be more effective in improving soil nutrients and reducing soil pH than other intercropping systems.

### 3.2 Composition and structures of the soil bacterial in different tea plant/legume intercropping systems

**Figure 1:**
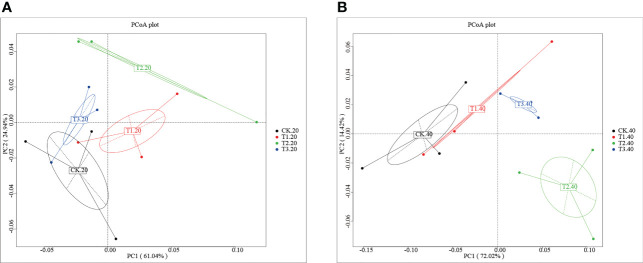
Principal coordinates analysis of the bacterial community in **(A)** 0-20 cm and **(B)** 20-40 cm soil of monocropping and intercropping systems.

Analysis of the core pan showed that the samples were enough to cover the abundance of species (**Supplementary Figure 1**). To investigate the differences number of genes between groups, a moderate divergence was observed between groups. As shown in **Supplementary Figure 2A** and **Supplementary Table 1**, the gene number ranged from 910,315 (CK-20-1) to 1,201,450 (T3-40-2). The Venn diagram showed that the four cropping systems separated clearly (**Supplementary Figure 2B**). T1 has the most significant number of unique genes, while the unique genes of T3 were fewest than that of other groups. To further explore the variation of soil bacterial communities under different intercropping systems, principal coordinates analysis (PCoA) at the phylum level was conducted (**Figure 1**). It was shown that the samples were separated from each other in both 0-20 and 20-40 cm soils.

**Figure 2:**
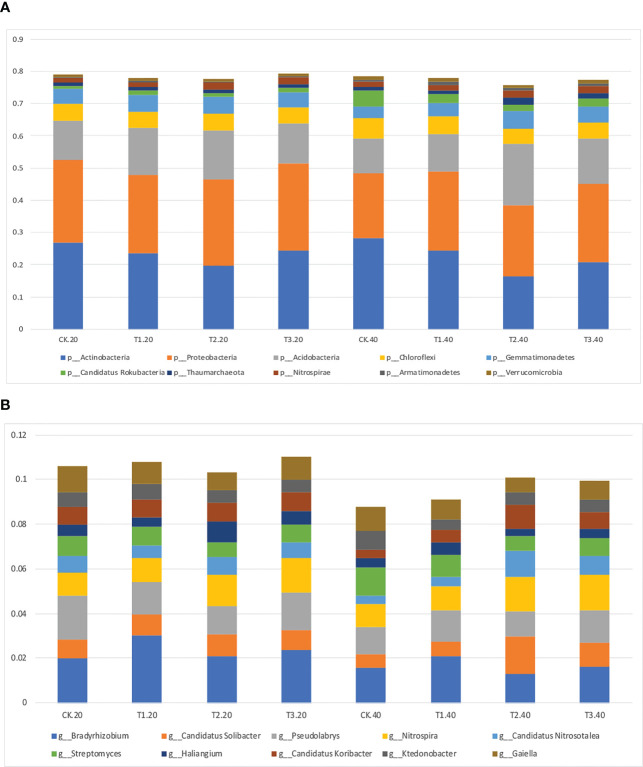
Relative abundance of top 10 bacterial **(A)** phyla and **(B)** genera in monocropping and intercropping systems.

Intercropping affected the diversity of soil microbes in both 0-20 and 20-40 cm soil. Actinobacteria was the most abundant bacterial phyla in monoculture, while Proteobacteria increased to the highest abundance phylum in intercropping systems (**Figure 2A**; **Supplementary Table 2**). It’s interesting to note that T2 had the lowest relative abundance of Actinobacteria, and T3 had the highest abundance of Proteobacteria.

The bacterial communities at the genus level were more diversified than those at the phylum level (**Figure 2B**; **Supplementary Table 3**). *Bradyrhizobium*, *Gaiella*, and *Pseudolabrys* were markedly enhanced in tea plant monoculture in 0–20 cm soils. However, in intercropping systems, *Bradyrhizobium*, *Nitrospira*, and *Pseudolabrys* had more significant relative abundances than those in the monoculture. Similar outcomes were seen in soils at 20–40 cm depths.

Among the bacterial species, the dominant species in 0-20 and 20-40 cm soil showed no significant differences, which the dominant species were *Actinobacteria bacterium* 13_1_20CM_4_69_9, *Actinobacteria bacterium* 13_2_20CM_68_14, and *Pseudolabrys* sp. Root1462, except *Candidatus Solibacter usitatus* in 20-40 cm soils of T3 (**Supplementary Figure 3** and **Supplementary Table 4**). However, the relative abundance of the dominant species was decreased in tea plant/legume intercropping compared with tea plant monoculture.

These taxonomic distributions suggest distinct bacterial community differences in soils between monocropping and intercropping systems and between different intercropping systems.

### 3.3 Relative abundance of the soil bacterial in different tea plant/legume intercropping systems

**Figure 3:**
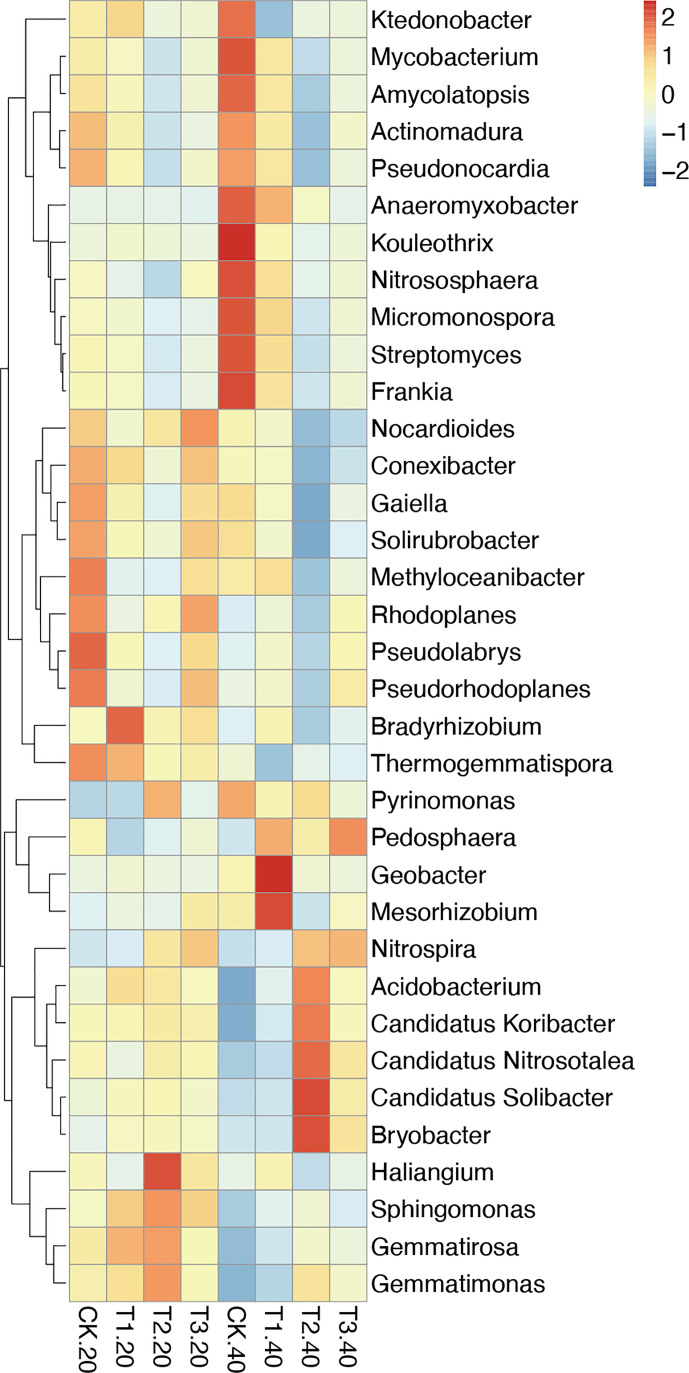
Relative abundance (Top 35) at the genus level in monocropping and intercropping soils. The abundances were normalized by Z-score. (CK: tea plant monocropping, T1: tea plant/mung bean intercropping, T2: tea plant/adzuki bean intercropping, T3: tea plant/mung bean and adzuki bean mixed intercropping, 20: 0-20 cm soil, 40: 20-40 cm soil).

The relative abundance of the top 35 most abundant phyla, genera, and species showed evident variations in the relative abundance of bacterial communities in different intercropping systems (**Figure 3**; **Supplementary Figure 4**). The relative abundance of the predominant genera varied amongst various intercropping systems. In 0-20 cm soil, *Bradyrhizobium*, *Sphingomonas*, and *Thermogemmatispora* considerably outperformed in T1, *Haliangium*, *Sphingomonas*, *Gemmatirosa*, and *Gemmatimonas* in T2, while *Nocardioides* and *Rhodoplanes* were more enriched in T3.

Compared with 0-20 cm soils, the bacterial communities showed more diversity in 20-40 cm soils. In detail, *Geobacter* and *Mesorhizobium*, which belong to Proteobacteria, were highly abundant in T1. T2 had greater abundances of *Candidatus Solibacter*, *Bryobacter*, *Candidatus Koribacter*, *Acidobacterium* (phylum Acidobacteria), and *Candidatus Nitrosotalea* (phylum Thaumarchaeota). The relative abundance of *Pedosphaera* and *Nitrospira* in T3 was higher than in other samples.

At the species level, *Bradyrhizobium elkanii* and *Haliangium ochraceum* were significantly enriched in 0-20 cm soils of T1 and T2, respectively. In 20-40 cm soil, several *Acidobacteriaceae* bacteria were abundant in T2, and *Rhodospirillales* bacterium URHD0088 (phylum Proteobacteria) was higher in T3 (**Supplementary Figure 4B**).

### 3.4 Biomarker bacterial taxa and functional analysis of soil bacterial in different tea plant/legume intercropping systems

**Figure 4:**
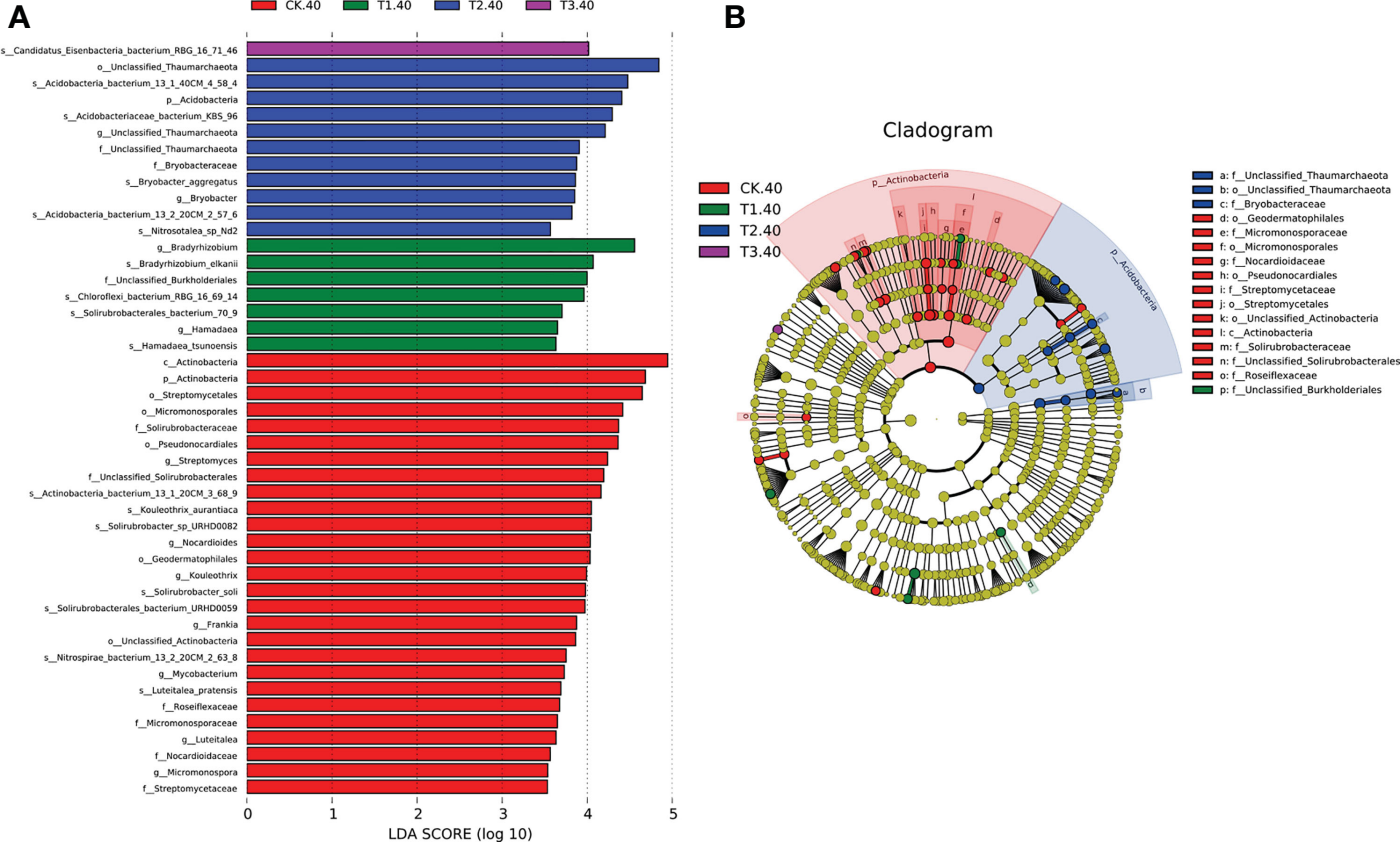
**(A)** LEfSe for bacterial taxa of 20-40 cm soils between monocropping and intercropping, **(B)** Cladogram showing significantly enriched bacterial taxa (from phylum to family level). Significant differences are defined at p < 0.05 and LDA score > 3.5 (CK: tea plant monocropping, T1: tea plant/mung bean intercropping, T2: tea plant/adzuki bean intercropping, T3: tea plant/mung bean and adzuki bean intercropping, 40: 20-40 cm soil).

The linear discriminant analysis with effect size (LEfSe) was employed to identify the differences in the bacterial community at different taxonomic levels between tea plant/legume intercropping systems. Based on our LDA score cut-off, some remarkable differences in bacterial species were evident between samples in different intercropping systems (**Figure 4**). In 20-40 cm soils, the abundance of *Bradyrhizobium elkanii* species was significantly higher in T1, followed by *Chloroflexi bacterium*_RBG_16_69_14 and *Solirubrobacterales bacterium*_70_9. *Actinobacteria bacterium* 13_1_40CM_4_58_4, *Actinobacteria bacterium* KBS_96, *Bryobactor aggregatus*, and *Acidobacteria bacterium* 13_2_20CM_2_57_6 were more abundant in T2. In addition, *Candidatus Eisenbacteria bacterium* RBG_16_71_46 was significantly higher in T3. Within the Actinobacteria phylum, f_Unclassified Thaumarchaeota and f_Bryobacteraceae were abundant in T2 (**Figure 4B**). However, in 0-20 cm soils, only *Solirubrobacterales bacterium* 70_9 was abundant in T3 (**Supplementary Figure 5**). Overall, LEfSe analysis identified 17 differential microbes between different intercropping systems that were selected for further analyses.

The KEGG functional enrichment identified 46 major level-2 sub-systems in the metagenome samples (**Supplementary Table 5**). Among the major level-2 sub-systems identified, carbohydrate metabolism, amino acid metabolism, energy metabolism, metabolism of cofactors and vitamins, and nucleotide metabolism were the top five level-2 sub-systems in all samples. The other significant pathways were translation, membrane transport, cellular community – prokaryotes, signal transduction, and replication and repair.

### 3.5 Changes of metabolites in different tea plant/legume intercropping systems

**Figure 5:**
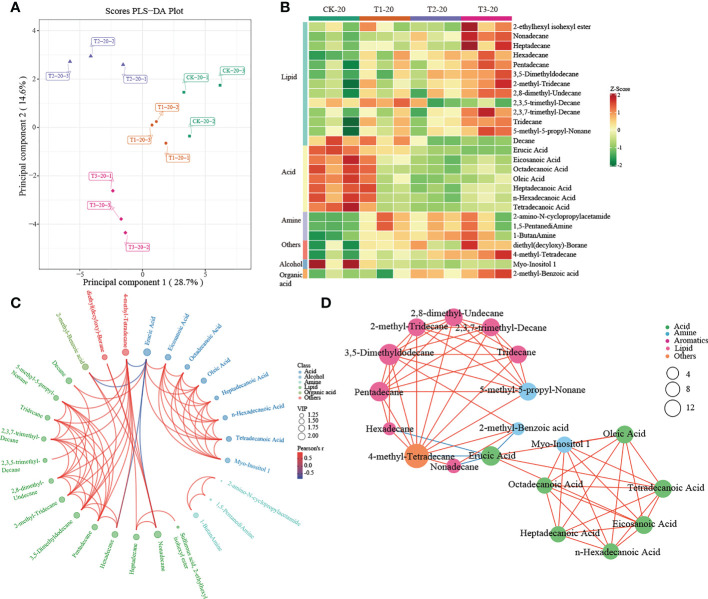
Metabolomics analysis of different cropping patterns in 0-20 cm soils. **(A)** PLS-DA score plots derived from metabolites at 0-20 cm soils, **(B)** heatmap analysis of the differential relative content of metabolites, chord diagram **(C)**, and co-occurrence network **(D)** of differently abundant metabolites. Variable Importance in Projection (VIP) scores of the significant differentially metabolites. Red lines indicate positive, and blue lines indicate negative correlations. The abundances were normalized by Z-score.

**Figure 6:**
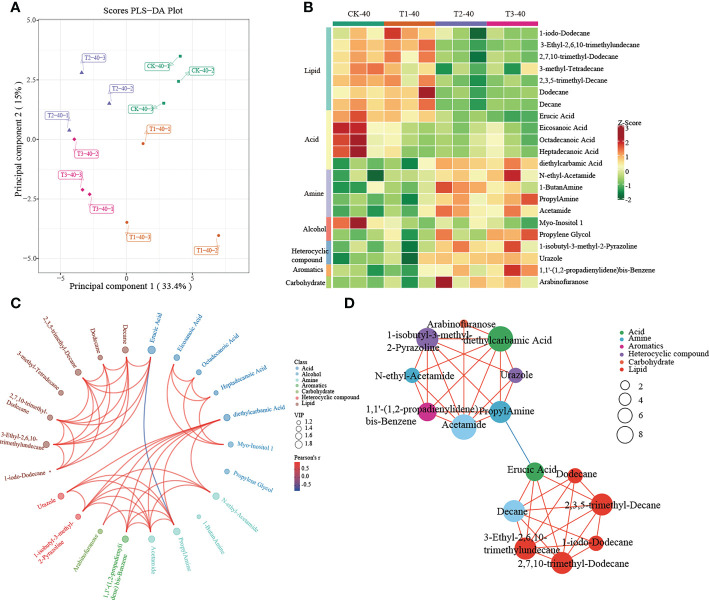
Metabolomics analysis of different cropping patterns in 20-40 cm soils. **(A)** PLS-DA score plots derived from metabolites at 20-40 cm soils, **(B)** heatmap analysis of the differential relative content of metabolites, chord diagram **(C)**, and co-occurrence network **(D)** of differently abundant metabolites. Variable Importance in Projection (VIP) scores of the significant differentially metabolites. Red lines indicate positive, and blue lines indicate negative correlations. The abundances were normalized by Z-score.

A multivariate analysis method, PLS-DA, was performed to investigate changes in soil metabolites under intercropping culture. Score plots showed that the metabolites in T1, T2, and T3 were statistically separated in both 0-20 and 20-40 cm soils (**Figures 5A**, **6A**). In total, 41 metabolites, 27 in 0-20 cm and 22 in 20-40 cm soil were differentially abundant between monocropping and different intercropping treatments (**Supplementary Tables 6**, **7**). The identified compounds include lipids, fatty acids, amine, alcohol, heterocyclic compounds, carbohydrate, aromatics, organic acids, and others. Specifically, lipids showed the most significant changes in both 0-20 cm and 20-40 cm soil, accounting for 48% (13/27) and 32% (7/22) of the total, respectively, followed by fatty acid and amine.

A heatmap was used to visualize the differentially expressed metabolites between different intercropping systems. In 0-20 cm soil, T1 mainly drives the accumulation of fatty acids (such as heptadecanoic acid, eicosanoic acid, octadecanoic acid, tetradecanoic acid, and so on) and lipids (decane). Much differently, lipids (such as 2-Ethylhexyl isohexyl ester, nonadecane, heptadecane, etc.), others (diethyl (decyloxy)-Borane and 4-methyl- tetradecane) and organic acid (2-methyl-Benzoic acid) were greatly increased in T3 (**Figure 5B**). In addition, the chord diagram results showed that 4-methyl-Tetradecane, significantly enriched in T3, was positively correlated with lipid while negatively correlated with Erucic Acid (**Figure 5C**). Similar results were observed in the co-occurrence network (**Figure 5D**). These results suggested that 4-methyl-Tetradecane may be the key differential metabolite affected by intercropping root interaction.

In 20-40 cm soil, there were no differences in metabolites between CK and T1, which were significantly enriched in lipid and fatty acids. In contrast, significant enrichment in amine, alcohol, heterocyclic compounds, aromatics, and carbohydrates was observed in T2 and T3 (**Figure 6B**). Diethylcarbamic Acid, Acetamide, and 1-isobutyl-3-methyl-2-Pyrazoline, which were significantly enriched in T2 and T3, were strongly correlated with other metabolites (**Figure 6C, D**).

Overall, the results demonstrated that intercropping, especially T2 and T3, could stimulate significant changes in metabolites in both 0-20 and 20-40 cm soils.

### 3.6 Correlations between the soil metabolism and bacterial community

**Figure 7:**
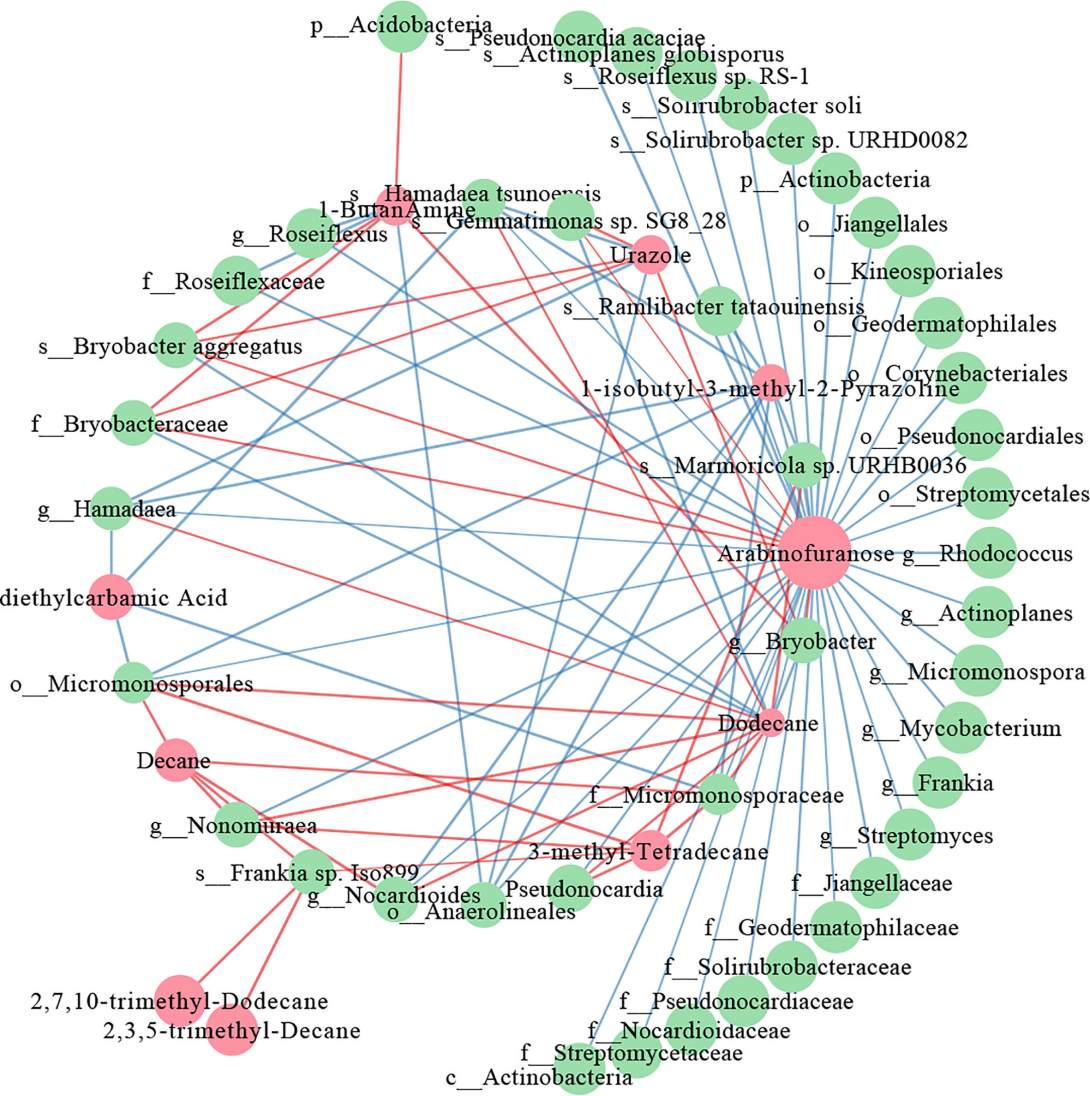
Co-occurrence network of the differential metabolites and differential bacterial taxa in 20-40 cm soils. The red nodes represent the differential metabolites, and the green nodes represent differential bacterial taxa. Red lines indicate positive, and blue lines indicate negative correlations.

Interactive networks were constructed to elucidate the relationship between the differential metabolites and the differential microorganisms in 0-20 and 20-40 cm soils. In the 20-40 cm soils, there were more positive correlations than negative correlations in the network within the metabolites, whereas the microbes showed the opposite results (**Figure 7**). The similar node size of soil microbes suggested that these microbes take part in the metabolism equivalently. For differential metabolites, arabinofuranose, which was abundant in tea plants/adzuki bean intercropping soils, showed the most remarkable correlation with the soil bacterial taxa, being especially positively correlated with the *Bryobacter aggregatus* species, Bryobacteraceae family (**Figures 4**, **7**). However, fewer differential metabolites and microbes exist in the co-occurrence network of 0-20 cm soils (**Supplementary Figure 6**). Eicosanoid acid positively correlated with Corynebacteriales order and *Mycobacterium* genus for differential metabolism. A positive correlation between myo-inositol 1 and the *Mycobacterium* genus was also detected.

## 4 Discussion

Intercropping can improve soil physicochemical characteristics and the microbial community due to interspecific differences and interactions in soil (Dai et al., 2019). This study examined the changes in the bacterial community and metabolites stemming from tea plant intercropping with different legume species. The analysis of bacterial and metabolism networks gives us a different understanding of the influence of intercropping on the soil.

### 4.1 Intercropping tea plants with different legumes increased the soil nutrients

Previous studies have demonstrated that intercropping systems can improve the content of various nutrients (Tang et al., 2021; Wang et al., 2022). Our results confirmed that a higher content of AK and TK and lower soil pH were detected in tea plant/legume intercropping than in monoculture tea plants. These results indicated that intercropping simultaneously could improve soil nutrient content, consistent with previous studies (Huang et al., 2022). Unlike many annual crops, the tea plant is a perennial crop that grows in acidic soil with an interval of pH 4.0 - 5.5 (Ruan et al., 2007; Yang et al., 2018). The high pH of the soil has always been an urgent problem in tea plantations in northern China. Our study demonstrated that intercropping could significantly reduce soil pH and increase DOC content, especially in 20-40 cm soils T3. These results might be attributed to tea plants having species-specific influences on each other when intercropping with different legume species (Tang et al., 2014). In this study, our data suggested that tea plants/adzuki beans and tea plants/adzuki bean and mung bean intercropping increased soil nutrients in 20-40 cm depth compared with 0-20 cm soils. The results were consistent with previous studies that the interactions between plants/legume roots could improve soil fertility since the roots of the adzuki bean were longer than the mung bean (Huang et al., 2022). In conclusion, these results suggested that intercropped tea plants and legumes have some advantages regarding soil nutrients.

### 4.2 Effect of intercropping on bacterial community diversity

Intercropping is expected to modify soil physicochemical properties, forcing a specific subset of the functional microbiome to enrich in soil, as evidenced by the increased microbial community diversity in the intercropping soil (Blaise et al., 2021; Bai et al., 2022). In this study, Proteobacteria, Actinobacteria, Acidobacteria, Nitrospira, Bacteroidetes, and Gemmatimonadetes were highly abundant bacterial phyla in different intercropping patterns, which is consistent with the results of previous studies investigating agricultural soils (Bai et al., 2022; Huang et al., 2022). We also observed that Proteobacteria increased to the highest abundance phylum in intercropping systems, especially in T3 (**Figure 2A**). It is well known that a wide range of nitrogen-fixing bacteria belong to Proteobacteria (Rahav et al., 2016; Li et al., 2019). According to these results, we hypothesized that legumes utilized these special bacterial groups to help themselves obtain the necessary N. Actinobacteria is the primary soil phylum, which plays an important role in nutrient cycling and organic matter degradation (Duran et al., 2015). In the LEfSe analysis of 20-40 cm soils, the abundance of Actinobacteria was more profound in monoculture than in intercropping soils (**Figure 4**). Compared with T1 and T2, only one species was more profound in T3. In addition, we observed a high abundance of organic matter and dissolved organic carbon in intercropping soils (**Table 1**). These results showed that the abundance of Actinobacteria was higher in soils with low organic matter and alkaline soils, which agreed with previous studies (Sun et al., 2022).

### 4.3 Correlation between the composition of soil microbes and soil metabolites

To further elucidate the relationship between the differential microbes and differential metabolites in intercropping systems, a spearman’s correlation analysis was constructed. The co-occurrence network revealed that carbohydrate (arabinofuranose) and lipids (2,7,10-trimethyl-Dodecane, 3-methyl-Tetradecane, 2,3,5-trimethyl-Decane, Dodecane, and Decane) shown frequent significant correlations with bacterial communities, indicating that the microbiota can interact with metabolites, or metabolites could activate them to improve soil fertility and change to adapt to environmental stress (**Figure 7**). These are consistent with the results of the metabolome analysis of 0-20 and 20-40 cm soils (**Figures 5**, **6**), demonstrating the vital role of lipids in improving soil nutrients of intercropping systems.

Previous studies reported that arabinofuranose was related to the carbon and nitrogen cycles and played essential roles in improving soil nutrients (Wu, 2021). Arabinofuranose was significantly enriched in tea plants/adzuki bean intercropping soils (**Figure 6**), suggesting that tea plants and adzuki intercropping could affect bacterial diversity through carbon and nitrogen cycles. It is known that intercropping could affect the distribution of bacterial communities *via* root interaction (Dong et al., 2022). However, the role of bacteria in connection with lipids remained poorly understood. Lipids are key compounds of the plasma membrane, as well as one category of the predominant soil metabolites that could facilitate the abiotic stress adaptation of plants (Wang et al., 2022). Interestingly, most lipids-related metabolites (4/5) were positively correlated with the Actinobacteria phylum (f_Micromonosporaceae, g_*Pseudonocardia*, g_*Nocardioides*, s_*Frankia*, and g_*Nonomuraea*) (**Figure 7**). These results indicated that the interaction of lipids and Actinobacteria might improve soil nutrition and bacterial composition in intercropping systems.

## 5 Conclusion

In summary, we investigated the effects of different species of legumes as intercropping plants in tea plantations. The bacteria related to carbohydrate metabolism and amino acid metabolism were significantly enriched. The differential metabolites of soils were enriched in lipids, fatty acids, amine, alcohol, and carbohydrate, and most of them showed positive correlations with each other. In particular, there was a positive correlation between lipids and Actinobacteria in the intercropping soils. The results revealed that tea plants/adzuki bean intercropping was more effective in increasing bacterial diversity and metabolite accumulation; tea plants/mung bean, adzuki bean mixed intercropping showed more advantages in decreasing soil pH and pressing weed. This study enhances our understanding of the ecological role of metabolites and microbes in the intercropping of tea plants and different species of legumes.

## Data availability statement

The original contributions presented in the study are publicly available. This data can be found here: NCBI, PRJNA892918.

## Author contributions

ZD and SW contributed the primary idea, analyzed most of the data, and wrote the initial draft of the paper. XZ, XL, JS, LS, SZ, and YW collected the soil samples. SW, XZ, and XL performed the soil properties measurement. All authors contributed to the article and approved the submitted version.
