# Supplementary material for: Different changes of bacterial diversity and soil metabolites in tea plants-legume intercropping systems
Source: Front Plant Sci. 2023 Mar 16;14:1110623. doi: 10.3389/fpls.2023.1110623 (PMC10060988; doi:10.3389/fpls.2023.1110623)
Supplement: Supplementary file 1 [file DataSheet_1.zip › Supplementary Figures.DOCX]

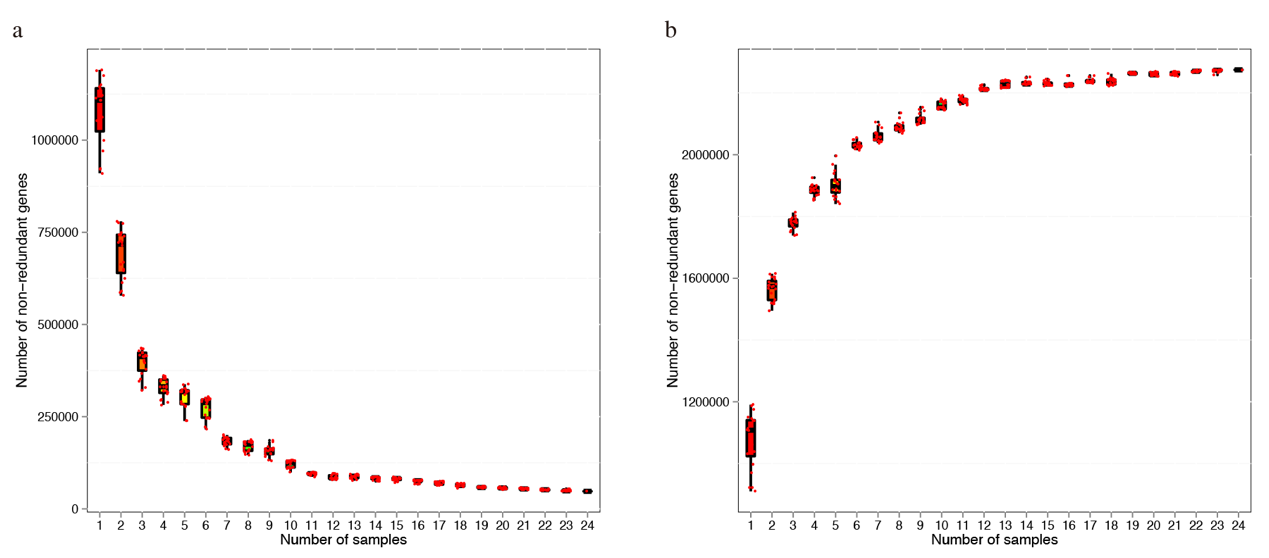


Figure S1 Core-pan gene dilution curves. a: core gene dilution curve, b: pan gene dilution curve.


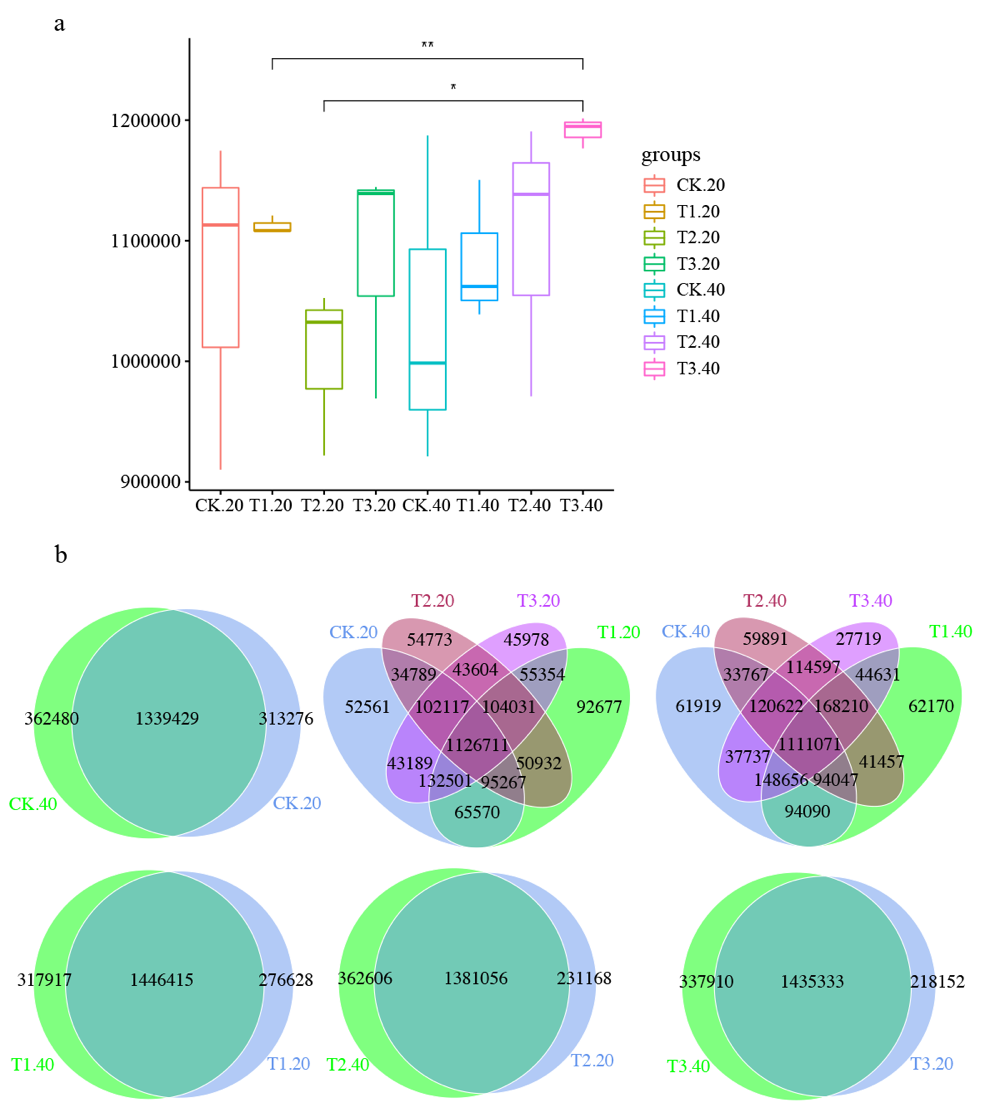


Figure S2 Different analysis of gene number. a: the box diagram of gene number between groups, b: the Venn graph of gene number between groups. CK: tea plant monocropping, T1: tea plant/mung bean intercropping, T2: tea plant/adzuki bean intercropping, T3: tea plant/mung bean and adzuki bean intercropping, “20” represents 0-20 cm soil core, “40” represents 20-40 cm soil core.


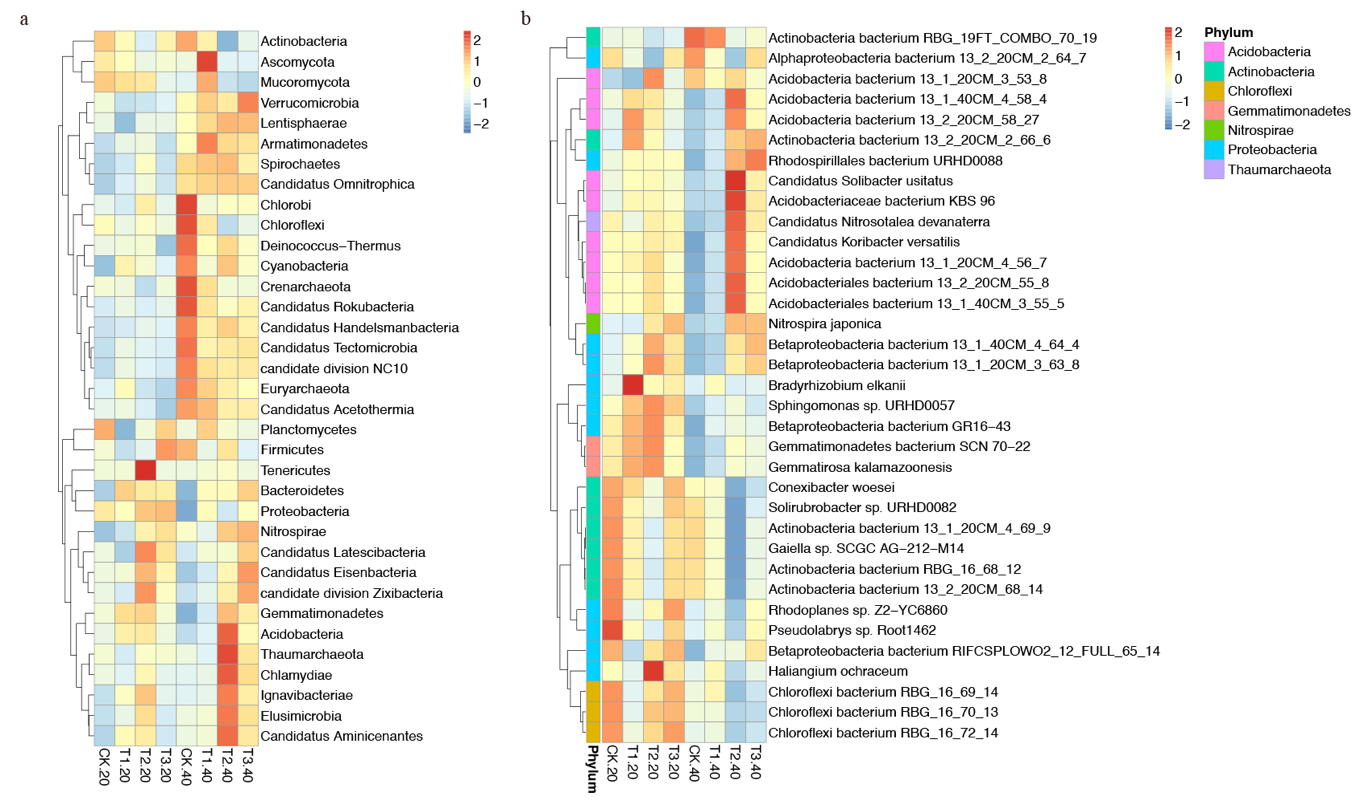
Figure S3 Relative abundance of top 10 bacterial species in monocropping and intercropping systems.

Figure S4 Relative abundant (Top 35) at the (a) phylum and (b) species level in monocropping and intercropping soils. The abundances were normalized by Z-score.


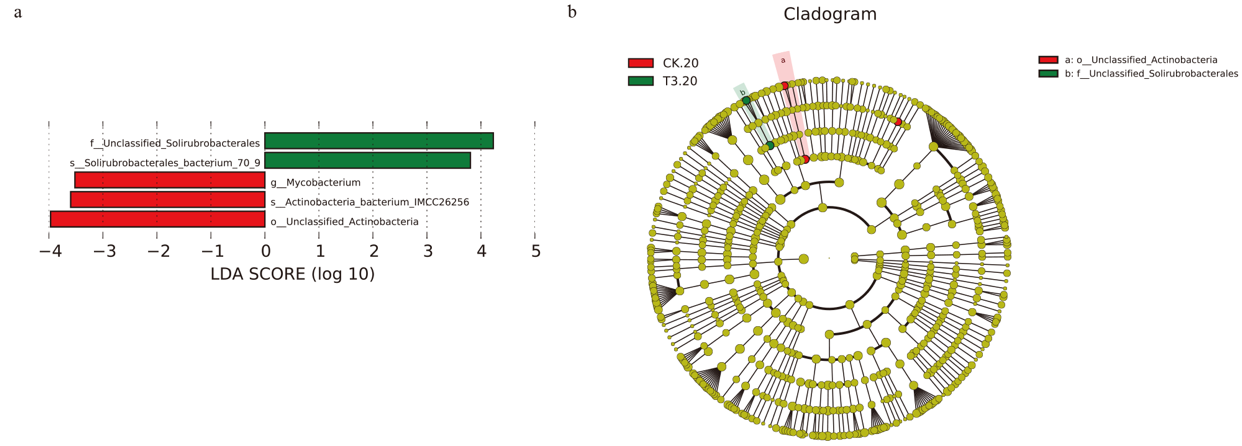


Figure S5 (a) LEfSe for bacterial taxa of 0-20 cm soils between monocropping and intercropping, (b) Cladogram showing significantly enriched bacterial taxa (from phylum to family level). Significant differences are defined at *p* < 0.05 and LDA score > 3.5.


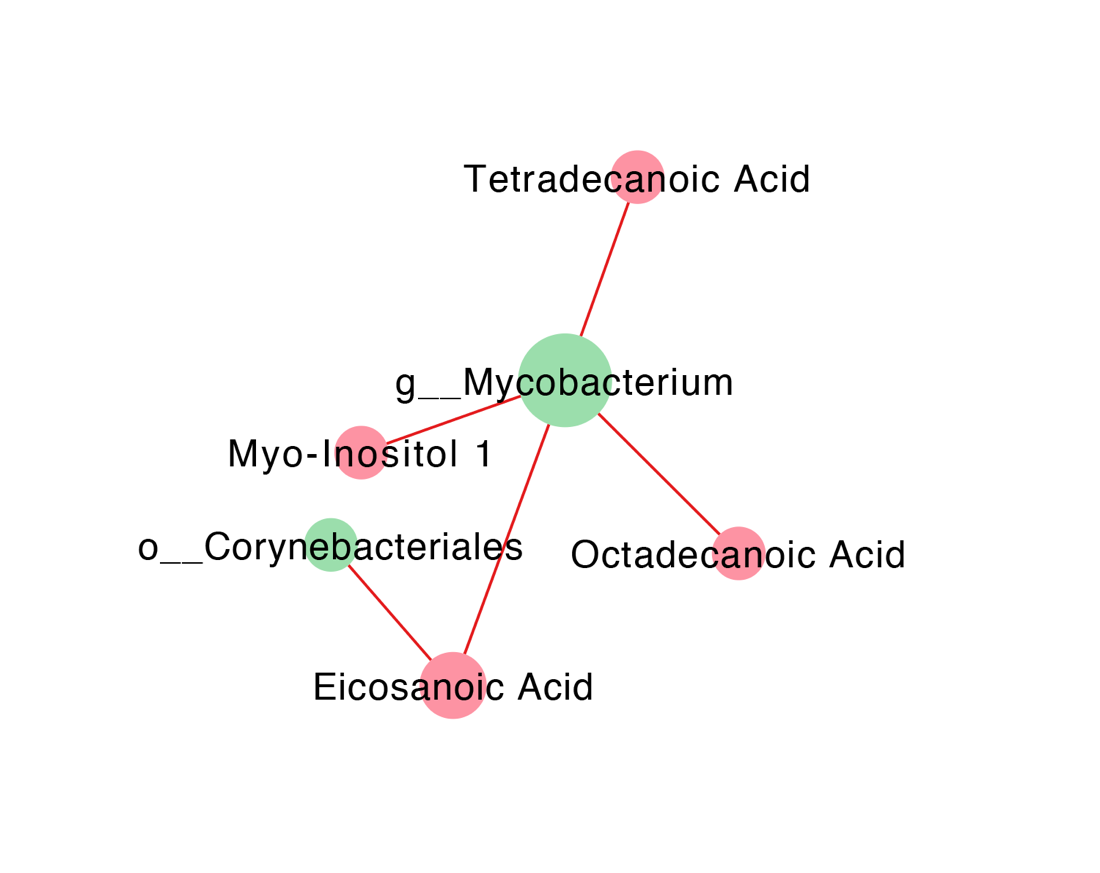


Figure S6 Co-occurrence network of the differential metabolites and differential bacterial taxa in 0-20 cm soils. The red nodes represent the differential metabolites, and the green nodes represent differential bacterial taxa. Red lines indicate positive, and blue lines indicate negative correlations.
